# Unexpectedly complex gradation of coral population structure in the Nansei Islands, Japan

**DOI:** 10.1002/ece3.2296

**Published:** 2016-07-12

**Authors:** Yuna Zayasu, Yuichi Nakajima, Kazuhiko Sakai, Go Suzuki, Noriyuki Satoh, Chuya Shinzato

**Affiliations:** ^1^ Marine Genomics UnitOkinawa Institute of Science and Technology Graduate University1919‐1 TanchaOnna‐sonOkinawa904‐0405Japan; ^2^ Marine Biophysics UnitOkinawa Institute of Science and Technology Graduate University1919‐1 TanchaOnna‐sonOkinawa904‐0405Japan; ^3^ Sesoko StationUniversity of the Ryukyus3422 SesokoMotobuOkinawa905‐0227Japan; ^4^ Ishigaki Tropical StationSeikai National Fisheries Research InstituteFisheries Research Agency148‐446 Fukai‐otaIshigakiOkinawa907‐0451Japan

**Keywords:** *Acropora tenuis*, gene flow, genetic diversity, microsatellite, population genetics

## Abstract

To establish effective locations and sizes of potential protected areas for reef ecosystems, detailed information about source and sink relationships between populations is critical, especially in archipelagic regions. Therefore, we assessed population structure and genetic diversity of *Acropora tenuis*, one of the dominant stony coral species in the Pacific, using 13 microsatellite markers to investigate 298 colonies from 15 locations across the Nansei Islands in southwestern Japan. Genetic diversity was not significant among sampling locations, even in possibly peripheral locations. In addition, our results showed that there are at least two populations of *A. tenuis* in the study area. The level of genetic differentiation between these populations was relatively low, but significant between many pairs of sampling locations. Directions of gene flow, which were estimated using a coalescence‐based approach, suggest that gene flow not only occurs from south to north, but also from north to south in various locations. Consequently, the Yaeyama Islands and the Amami Islands are potential northern and southern sources of corals. On the other hand, the Miyako Islands and west central Okinawa Island are potential sink populations. The Kerama Islands and the vicinity of Taketomi Island are potential contact points of genetic subdivision of coral populations in the Nansei Islands. We found that genetic population structure of *A. tenuis* in the Nansei Islands is more complex than previously thought. These cryptic populations are very important for preserving genetic diversity and should be maintained.

## Introduction

Coral reefs are highly productive ecosystems that provide habitat for a great variety of marine organisms. Humans derive many benefits from them, including fisheries and tourism (Cesar et al. 2003). Despite their importance, coral reefs are threatened globally by climate change and anthropogenic influences. Understanding population structure is essential for effective reef management and restoration of damaged reefs (reviewed in West and Salm 2003). Because successful recruitment is fundamental to the resilience of coral populations (Richmond 1997; Ritson‐Williams et al. 2009), knowledge of source and sink dynamics is essential to create effective Marine Protected Areas and to predict large‐scale effects of habitat changes, especially within oceanic archipelagic systems (Nakajima et al. 2010; Polato et al. 2010; Golbuu et al. 2012; Davies et al. 2015; Shinzato et al. 2015). Furthermore, while transplantation of coral fragments has been one of the most frequently recommended approaches to enhance coral abundance on degraded reefs (Omori and Fujiwara 2004; Rinkevich 2008; Young et al. 2012), there have been concerns about adverse effects of transplantation, as there have been in regard to tree transplantation (Keller et al. 2000; Edmands and Timmerman 2003), an analogous situation. These concerns include reduced genetic diversity, breakdown of local genetic structure, and genetic introgression (Omori and Fujiwara 2004; Baums 2008).

The Nansei Islands (Nansei) are a 1200 km chain of approximately 200 islands that exhibit high levels of biodiversity and endemism (Itô et al. 2000; Kuo et al. 2006; Lucifora et al. 2011). These subtropical to temperate islands range from 24° to 31° north latitude (Fig. 2A). The Nansei are divided into six islands groups: from northeast to southwest, the Osumi, Tokara, Amami, Okinawa, Sakishima, and Daito Island groups the latter being located east of the Okinawa group (Fig. 2B). The Sakishima group is subdivided into three subgroups: the Miyako, Yaeyama, and Senkaku Islands (Ajiro and Warita 2009; Fig. 2B). The Kuroshio Current, which is one of the strongest warm currents in the world, flows northeastward into the East China Sea, passing along the continental slope east of Taiwan and through the Tokara Strait (Fig. 2A). Among the world's coral reefs, the Nansei are one of the richest centers of endemism and one of the highest priority conservation areas (Roberts et al. 2002).

The Nansei experienced extensive decimation bleaching at the time of the 1998 global coral bleaching event (Goreau et al. 2000; Loya et al. 2001). Moreover, most reefs in this region have been threatened by repeated outbreaks of crown‐of‐thorns starfish (Yamaguchi 1986). Some studies report that terrestrial runoff from large‐scale agriculture, land development, and other anthropogenic disturbances have also impeded recovery (Hongo and Yamano 2013).

**Figure 1:**
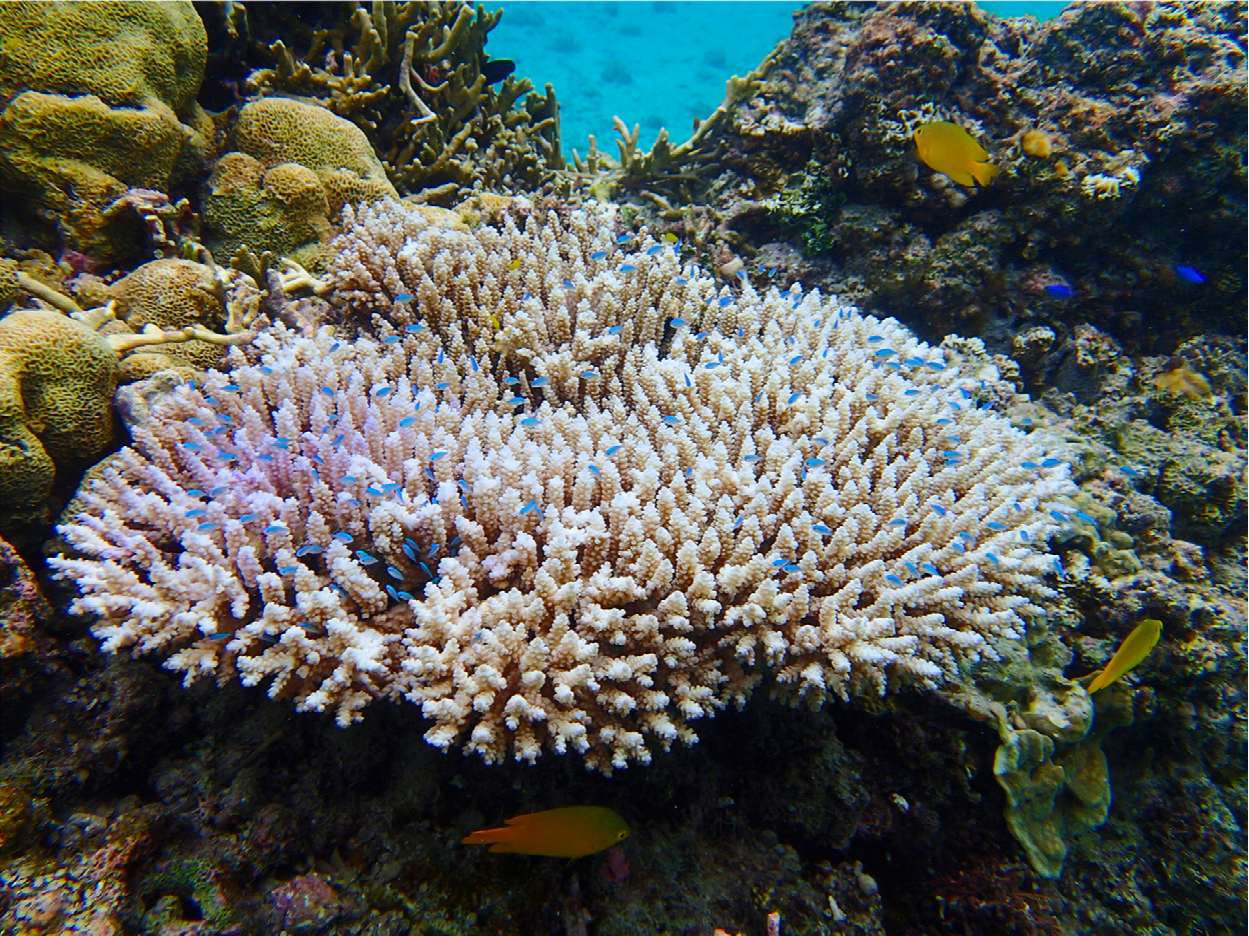
A colony of *Acropora tenuis* at the population Shigira, Miyako Island.

The Genus *Acropora* is the largest genus of reef‐building corals, both in terms of distribution and species richness (Wallace 1999). The complex structures of acroporid corals provide habitat and refuge for more than a million species of marine organisms (Hinrichsen 1997). The corymbose coral, *Acropora tenuis* (Dana, 1846; Fig. 1), is common in middle depths of reef slopes in the western Pacific and the Red Sea (Veron 2000; Suzuki et al. 2008). *Acropora tenuis* is a hermaphroditic broadcast spawner, with an annual gametogenic cycle. Early lifestages of this species have been studied in various locations owing to its habit of spawning earlier than other mass‐spawning acroporids (Fukami et al. 2003) and its larger number of eggs per polyp (Ohya and Iwao 1998). Furthermore, attempts at restoration and transplantation have been conducted using *A. tenuis* in Okinawa, Japan (Omori 2005; Omori et al. 2008).

In Japan, coral larvae are generally believed to recruit from south to north due to the Kuroshio Current (Veron 1992; Veron and Minchin 1992), an idea seemingly supported by recent studies suggesting that global warming has induced poleward shifts of coral reefs (Yamano et al. 2011; Yara et al. 2011). The Kerama Islands have been suggested as a source of coral larvae for Okinawa (Kimura et al. 1992; Nadaoka et al. 2002; Nishikawa 2008). However, there is no clear relationship between acroporid coral coverage in the Kerama Islands and the number of settled acroporid larvae on the west coast of Okinawa (Iwata and Sakai 2010). The center of the present axis of the Kuroshio Current is approximately 130–150 km west of Okinawa (Iwata and Sakai 2010); therefore, its influence on Okinawa appears indirect. Along west central Okinawa Island, quantitative observations and surface currents have suggested that factors other than the Kuroshio Current are involved in recruitment patterns of corals and predatory crown‐of‐thorns starfish (Nakamura et al. 2015). Recent analyses using whole‐genome, single nucleotide polymorphisms (SNPs) of a congeneric species, *Acropora digitifera* (Dana, 1846), suggested that the Kerama Islands are a historical sink population, but not a source population for Okinawa Island, the mere 30‐km distance separating them notwithstanding (Shinzato et al. 2015). However, the study area in the latter research was limited to the southern Nansei.

For effective restoration of coral reefs in the Nansei, extensive studies across habitats are needed. With this purpose, this study assessed reef connectivity and genetic diversity across the Nansei in Okinawa Prefecture, Japan. Specifically, we sought to ascertain whether gene flow occurs from the southern Yaeyama Islands to the northern Nansei, a distance of more than 1000 km. We also wanted to determine whether there exist sink populations for key reef‐building species other than *A. digitifera*, and if so, which islands serve this function. To answer these questions, using microsatellite markers, we studied another ecologically important species, *A. tenuis,* from a much broader range of localities than previous studies.

## Materials and Methods

### Sample collection

**Figure 2:**
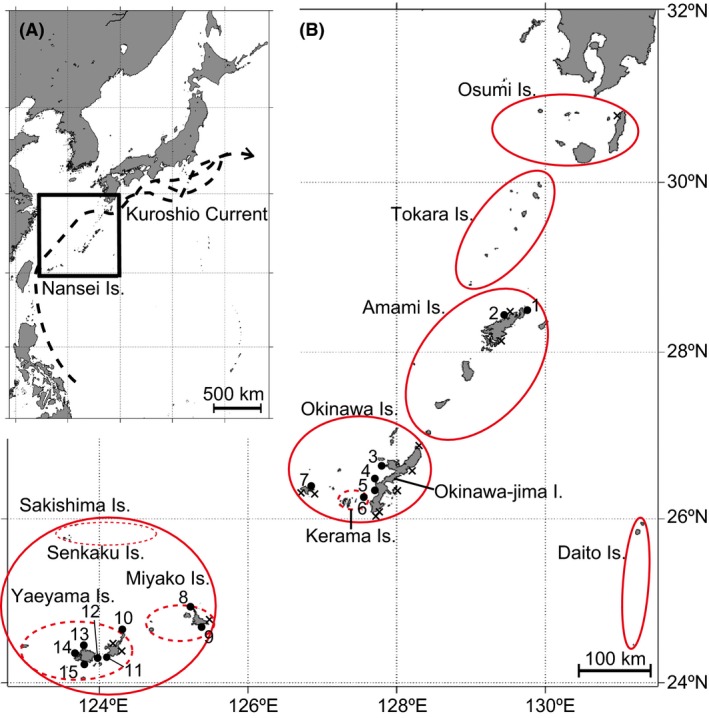
Map of the study area. (A) A map of east Asia, including the Nansei Islands, Japan (solid square). Dashed arrows represent the Kuroshio Current. (B) A map of the Nansei Islands, Japan. Red circles represent island groups, based on data from the Hydrographic and Oceanographic Department of the Japan Coast Guard. Dashed red circles represent Yaeyama subgroups. Populations (with names and sequential numbers) with ≥10 colonies of *Acropora tenuis* were used for analyses. Numbers correspond to Table 1. Populations marked with an × had fewer than 10 colonies of *A. tenuis*. These were not used for analyses.

From May 2014 to April 2015, and with permits from the Kagoshima and Okinawa Prefectural Governments, we conducted surveys at 28 locations in the Nansei that had high *Acropora* coverage (Table 1, Fig. 2B). An approximately 2‐cm branch fragment was collected from each coral colony at depths shallower than 10 m. At each sampling location, we collected fragments from all colonies of *A. tenuis* within approximately 3 ha, using snorkeling or SCUBA diving. Coral fragments were preserved in 99% ethanol and then brought to the laboratory for analysis. The 15 sampling locations that had 10 and more colonies of *A. tenuis* were analyzed as follows.

**Table 1:** Sampling location information and numbers of individuals sampled

Island group	Location	*N*	Locality	Latitude	Longitude	Geographical structure
Osumi		0	Urata, Tanegashima Island, Kagoshima Prefecture	30.825	131.038	Inside the Bay
Amami	1 Ayamaru	15	Ayamaru Cape, Amami‐oshima I., Kagoshima Pref.	28.474	129.716	Reef front
		1	Ohama Beach, Amami‐oshima I., Kagoshima Pref.	28.407	129.454	Reef front
	2 Kuninao	28	Kuninao Beach, Amami‐oshima I., Kagoshima Pref.	28.374	129.404	Reef front
		1	Yadori Beach, Amami‐oshima I., Kagoshima Pref.	28.121	129.362	Reef front
Okinawa		2	Oku Beach, Okinawa‐jima I., Okinawa Pref.	26.849	128.289	Reef front
	3 Sesoko	16	East of Sesoko‐jima I., Okinawa‐jima I., Okinawa Pref.	26.632	127.864	Reef front
		4	Oura Bay, Okinawa‐jima I., Okinawa Pref.	26.540	128.077	Reef front
	4 Maeda	12	Maeda Cape, Okinawa‐jima I., Okinawa Pref.	26.444	127.773	Reef front
		0	Miyagi‐Jima I., Okinawa‐jima I., Okinawa Pref.	26.367	127.995	Back reef moat
		0	Yaese‐cho, Okinawa‐jima I., Okinawa Pref.	26.108	127.740	Reef front
		2	Odo Beach, Okinawa‐jima I., Okinawa Pref.	26.085	127.701	Reef front
	5 Isa	16	Isa, Okinawa‐jima I., Okinawa Pref.	26.296	127.744	Reef front
	6 Chibishi	17	Kami‐shima I., Chibishi, Kerama Is., Okinawa Pref.	26.267	127.575	Reef front
	7 Kume	12	North of Hatenohama Beach, Kume‐jima I., Okinawa Pref.	26.356	126.877	Reef front
		7	Ara Beach, Kume‐jima I. Okinawa Pref.	26.312	126.771	Back reef moat
		5	Takenchi, Kume‐jima I., Okinawa Pref.	26.321	126.857	Reef front
Miyako	8 Ikema	13	Ikema‐jima I., Miyako‐jima I., Okinawa Pref.	24.932	125.233	Reef front
		0	Yoshino Beach, Miyako‐jima I., Okinawa Pref.	24.748	125.444	Back reef moat
	9 Shigira	10	Shigira Bay, Miyako‐jima I., Okinawa Pref.	24.719	125.342	Inside the Bay
Yaeyama	10 Hirakubo	34	Hirakubo, Ishigaki‐jima I., Okinawa Pref.	24.609	124.326	Reef front
		3	Fukai, Ishigaki‐jima I., Okinawa Pref.	24.452	124.173	Back reef moat
		0	East of Ishigaki Airport, Ishigaki‐jima I., Okinawa Pref.	24.397	124.263	Reef front
	11 Taketomi	20	North of Taketomi‐jima I., Okinawa Pref.	24.342	124.094	Lagoon
	12 Kuroshima	16	North of Kuroshima I., Okinawa Pref.	24.301	124.016	Lagoon
	13 Nakano	30	Nakano Beach, Iriomote‐jima I., Okinwa Pref.	24.431	123.790	Reef front
	14 Amitori	39	Amitori Bay, Iriomote‐jima I., Okinawa Pref.	24.332	123.696	Inside the Bay
	15 Haemida	20	Haemida, Iriomote‐jima I., Okinawa Pref.	24.269	123.830	Reef front

Location names with sequential numbers had 10 and more colonies of *Acropora tenuis* were used for analyses. *N* represents the sample size per site.

### DNA extraction and microsatellite analyses

For PCR, genomic DNA was extracted from coral fragments using DNeasy Blood & Tissue Kits (QIAGEN, Hilden, Germany) and quantified using a NanoDrop™ 1000 Spectrophotometer (Thermo Fisher Scientific, Waltham, MA, USA). Universal primer sets for 13 microsatellite loci, developed for the Genus *Acropora* (Shinzato et al. 2014), were used for amplification of alleles. Four loci (4546m2, 8499m4, 7203m5, and 11401m4) were amplified under the following conditions. Each reaction mixture contained 10 ng/μL template DNA, AmpliTaq Gold 360 Master Mix (QIAGEN), and three primers for each locus: a nontailed reverse primer (0.5 μL), a forward primer with an M13 reverse (5′‐CAGGAAACAGCTATGAC‐3′) sequence tail (1 μL) and an M13 reverse primer (0.5 μL), fluorescently labeled with 6‐FAM, and MilliQ water (Merck Millipore, Darmstadt, Germany) to final volume of 10 μL. PCR cycling conditions consisted of an initial denaturation of 10 min at 95°C, followed by 32 cycles each of 30 sec at 95°C, 30 sec at 52°C (all loci), and 30 sec at 72°C, with an extension of 1 min at 72°C after the final cycle.

We also conducted PCR using a multiplexed approach, based on the primers of Shinzato et al. (2014). As forward primers, we designed 12406m3, 7961m4, and 11292m4 with the CAG tag (5′‐CAGTCGGGCGTCATCA‐primer; Hauswaldt and Glenn 2003), and 11543m5, 12130m5, and 11745m3 with the T7 terminator (5′‐CTAGTTATTGCTCAGCGGT). Each of these sequences was tagged at the 5′ end with a different color of fluorescent label (6‐FAM/blue, T7/green, or CAG/red). Three multiplex sets were used successfully (Appendix S1). Multiplexing was conducted in 10 μL reaction volumes using the QIAGEN PCR Multiplex Kit as follows: 2.5 μL Multiplex PCR Master Mix, 0.05 μL reverse primer, 0.05 μL labeled tail primer, 0.05 μL forward tailed primer, 1.05 μL MilliQ water, and 1 μL template DNA. PCR cycling conditions consisted of an initial denaturation for 15 min at 95°C, followed by 30 cycles each of 30 sec at 94°C, 45 sec at 57°C, 45 sec at 72°C, and then with 8 cycles each of 30 sec at 94°C, 45 sec at 53°C, and 45 sec at 72°C, with a final extension of 10 min at 72°C.

Allelic variations of PCR products were analyzed with Applied Biosystems 3130 and Applied Biosystems 3730*xl* Genetic Analyzers (Thermo Fisher Scientific). Fragment sizes were determined using GeneMapper software version 5.0 (Thermo Fisher Scientific) by comparison with a GeneScan™ 500 LIZ® (Thermo Fisher Scientific) internal lane size standard.

### Genetic diversity

The following parameters were calculated using FSTAT 2.9.3 software (Goudet 1995): numbers of alleles per locus, allelic richness (Ar) and inbreeding coefficients (*F*_IS_), observed heterozygosity (*H*_o_), expected heterozygosity (*H*_e_), and Hardy–Weinberg equilibrium. Evidence and frequency of null alleles were tested using Micro‐Checker v 2.2.3 (van Oosterhout et al. 2004), and levels of genotypic disequilibrium were computed using GENEPOP (Raymond and Rousset 1995). Pairwise population *F*_ST_ values were calculated, and analysis of molecular variance (AMOVA; Excoffier et al. 1992) was performed using GenAlex version 6.5 (Peakall and Smouse 2012). Statistical analyses were performed using MATLAB R2014a (MathWorks, Natick, MA, USA).

### Population structure

We examined population structure using Bayesian methods in STRUCTURE (Pritchard et al. 2000). No prior information regarding sampling locations was used in the admixture model with correlated allele frequency (Falush et al. 2003). Each run comprised a burn‐in period of 100,000 replications and a run length of 1,000,000 Markov chain Monte Carlo iterations. We then used the method of Evanno et al. (2005) using STRUCTURE HARVESTER (Earl and Vonholdt 2012), to infer the most appropriate number of genetic clusters (*K* value). CLUMPP (Jakobsson and Rosenberg 2007) was used to combine the output of 20 iterations from STRUCTURE at the appropriate *K* value.

The relationship between genetic differentiation and geographic distance was assessed for all pairwise comparisons between populations. We used a natural log scale of the Euclidean distance as the geographic distance and linearized *F*_ST_ as genetic distance. Analysis of isolation by distance was carried out using Mantel's test in GenAlex.

### Migration rate

The program MIGRATE‐N 3.6.11 (Beerli and Palczewski 2010) was used to estimate gene flow between populations. This coalescence‐based program estimated the mutation‐scaled migration rates *M* = *m*/*μ*, where *m* was the immigration rate per generation among sampling locations, and *μ* was the mutation rate per generation per locus. Migration rates among populations were performed using Bayesian inference and the Brownian motion mutation model. Most run parameters were left at default values.

### Ocean currents

We downloaded ocean current data from the J‐DOSS website of the Japan Oceanographic Data Center (http://www.jodc.go.jp/jodcweb/JDOSS/index_j.html). Then, data were averaged from waters shallower than 40 m, each May from 1990 to 2011, using a 0.5° grid. Current vector for velocity and direction were developed using SAGA software (System for Automated Geoscientific Analyses) version 2.2.0 (Conrad et al. 2015) and were then converted into grids and motion vectors for Google Earth V7.1.5.1557.

## Results

### Clonal structure, genetic diversity, and distribution

Genotyping of 13 microsatellite loci from 298 colonies at 15 sampling locations, revealed 298 genotypes, strongly suggesting that there are no clonal colonies of *A. tenuis* in the Nansei, within the region surveyed. Null alleles were detected in samples from several sites and loci, especially loci 7961m4 and 4546m2 (Table 2, Appendix S2). Significant linkage disequilibrium was not detected between pairs of loci. The genetic diversity parameters, Ar and *H*_e_, did not differ significantly among sampling locations (Kruskal–Wallis test; Ar, *P* = 0.9961; *H*_e_, *P* = 0.9990; Appendix S3). We also found that this species tends to be distributed along the northwestern coasts of islands, despite the fact that other *Acropora* species are normally distributed along southeastern coasts (Table 1, Fig. 2B).

**Table 2:** Number of alleles, observed (*H*_o_) and expected (*H*_e_) heterozygosity, and inbreeding coefficients (*F*_IS_) for each locus and location

	Locus	8346m3	7961m4	11745m3	12406m3	11543m5	530m4	11401m4	441m6	11292m4	8499m4	7203m5	12130m5	4546m2
Ayamaru	Number of alleles	6	2	11	6	4	5	6	5	4	4	6	2	3
	*H*_o_	0.733	0.067	1.000	0.667	0.733	0.867	0.867	0.667	0.467	0.667	0.400	0.333	0.133
	*H*_e_	0.729	0.358	0.891	0.773	0.611	0.696	0.769	0.718	0.576	0.507	0.531	0.278	0.424
	*F*_IS_	−0.006	0.814	−0.122	0.138	−0.200	−0.246	−0.127	0.071	0.189	−0.316	0.247	−0.200	0.686
Kuninao	Number of alleles	5	4	12	6	4	6	7	4	6	6	5	6	9
	*H*_o_	0.536	0.071	1.000	0.643	0.893	0.714	0.857	0.714	0.357	0.714	0.429	0.500	0.179
	*H*_e_	0.612	0.612	0.886	0.789	0.630	0.612	0.793	0.693	0.596	0.513	0.591	0.524	0.499
	*F*_IS_	0.125	0.883*	−0.128	0.185*	−0.417*	−0.167	−0.080	−0.030	0.400*	−0.393	0.274	0.045*	0.642*
Sesoko	Number of alleles	7	5	11	6	2	4	7	4	4	5	7	3	4
	*H*_o_	0.938	0.125	0.938	0.938	0.563	0.563	0.813	0.750	0.500	0.688	0.563	0.625	0.313
	*H*_e_	0.766	0.486	0.879	0.762	0.451	0.607	0.764	0.674	0.627	0.504	0.543	0.447	0.521
	*F*_IS_	−0.224	0.743*	−0.067	−0.231	−0.247	0.074	−0.064	−0.113	0.202	−0.364	−0.036	−0.397	0.401
Maeda	Number of alleles	7	3	13	5	2	4	7	3	7	3	5	4	4
	*H*_o_	0.833	0.000	0.833	0.750	0.250	0.500	0.500	0.333	0.583	0.167	0.583	0.750	0.167
	*H*_e_	0.740	0.611	0.899	0.767	0.469	0.413	0.785	0.642	0.691	0.292	0.649	0.521	0.413
	*F*_IS_	−0.127	1.000*	0.073	0.023	0.467	−0.210	0.363	0.481	0.156	0.429	0.102	−0.440	0.597*
Kume	Number of alleles	5	4	9	5	2	3	5	4	5	2	5	2	5
	*H*_o_	0.750	0.000	0.750	0.750	0.417	0.500	0.750	0.917	0.333	0.417	0.667	0.167	0.417
	*H*_e_	0.667	0.625	0.851	0.767	0.497	0.538	0.733	0.726	0.361	0.330	0.694	0.153	0.472
	*F*_IS_	−0.125	1.000	0.118	0.023	0.161	0.071	−0.024	−0.263	0.077	−0.263	0.040	−0.091	0.118
Isa	Number of alleles	6	4	10	6	2	6	6	5	3	2	6	2	4
	*H*_o_	0.500	0.125	0.750	0.625	0.625	0.625	0.625	0.938	0.563	0.375	0.563	0.500	0.125
	*H*_e_	0.609	0.549	0.834	0.779	0.500	0.598	0.729	0.674	0.537	0.375	0.746	0.375	0.229
	*F*_IS_	0.179	0.772*	0.101	0.198	−0.250	−0.046	0.142	−0.391	−0.047	0.000	0.246	−0.333	0.453
Chibishi	Number of alleles	5	4	16	7	2	4	6	4	4	3	6	3	5
	*H*_o_	0.706	0.176	0.941	0.824	0.235	0.235	0.706	0.412	0.471	0.529	0.412	0.471	0.118
	*H*_e_	0.727	0.559	0.905	0.811	0.415	0.528	0.720	0.649	0.528	0.476	0.711	0.372	0.396
	*F*_IS_	0.029	0.684	−0.040	−0.015	0.433	0.554	0.019	0.365	0.108	−0.113	0.421	−0.265	0.703
Ikema	Number of alleles	6	5	13	5	2	4	8	5	5	4	6	3	4
	*H*_o_	0.692	0.231	0.846	0.769	0.308	0.615	1.000	0.769	0.538	0.846	0.692	0.231	0.154
	*H*_e_	0.710	0.438	0.899	0.722	0.473	0.553	0.763	0.731	0.536	0.565	0.754	0.210	0.388
	*F*_IS_	0.025	0.473	0.059	−0.066	0.350	−0.112	−0.310	−0.053	−0.006	−0.497	0.082	−0.099	0.603
Shigira	Number of alleles	5	3	7	5	2	2	6	5	4	3	5	3	5
	*H*_o_	0.500	0.200	0.900	0.800	0.500	0.600	0.800	0.600	0.300	0.600	0.500	0.400	0.200
	*H*_e_	0.705	0.185	0.830	0.715	0.495	0.420	0.670	0.705	0.655	0.445	0.595	0.335	0.545
	*F*_IS_	0.291	−0.081	−0.084	−0.119	−0.010	−0.429	−0.194	0.149	0.542	−0.348	0.160	−0.194	0.633
Hirakubo	Number of alleles	4	4	14	7	3	4	6	5	6	2	9	3	7
	*H*_o_	0.706	0.088	0.912	0.882	0.235	0.529	0.676	0.559	0.324	0.441	0.735	0.324	0.235
	*H*_e_	0.724	0.510	0.895	0.782	0.501	0.593	0.734	0.707	0.495	0.375	0.718	0.279	0.538
	*F*_IS_	0.026	0.827*	−0.018	−0.129	0.531*	0.107	0.079	0.209	0.347	−0.176	−0.024*	−0.160	0.563*
Nakano	Number of alleles	7	6	15	7	3	7	6	3	6	6	8	3	6
	*H*_o_	0.733	0.167	0.767	0.733	0.300	0.600	0.667	0.600	0.467	0.733	0.800	0.300	0.233
	*H*_e_	0.753	0.388	0.897	0.782	0.513	0.630	0.736	0.640	0.602	0.528	0.731	0.316	0.324
	*F*_IS_	0.027	0.570*	0.146	0.063	0.415	0.048	0.094	0.063	0.224*	−0.389	−0.095	0.051	0.281
Taketomi	Number of alleles	4	5	13	5	3	3	4	5	3	3	7	3	5
	*H*_o_	0.550	0.050	0.900	0.900	0.450	0.500	0.850	0.700	0.100	0.450	0.650	0.250	0.150
	*H*_e_	0.648	0.654	0.898	0.766	0.499	0.521	0.636	0.654	0.335	0.411	0.671	0.226	0.453
	*F*_IS_	0.151	0.924	−0.003	−0.175	0.098	0.041	−0.336	−0.071	0.701	−0.094	0.032	−0.105	0.669
Amitori	Number of alleles	7	8	18	7	5	6	7	5	6	3	10	5	10
	*H*_o_	0.641	0.231	0.846	0.872	0.641	0.538	0.487	0.692	0.282	0.667	0.538	0.385	0.333
	*H*_e_	0.652	0.661	0.916	0.768	0.544	0.583	0.583	0.696	0.298	0.469	0.702	0.371	0.486
	*F*_IS_	0.017	0.651*	0.076	−0.136	−0.179	0.076	0.165*	0.006	0.053	−0.421	0.232	−0.036	0.314*
Kuroshima	Number of alleles	6	5	13	7	2	3	4	4	5	2	5	2	6
	*H*_o_	0.750	0.125	0.813	0.688	0.250	0.750	0.813	0.500	0.500	0.313	0.375	0.125	0.250
	*H*_e_	0.756	0.416	0.898	0.793	0.469	0.570	0.689	0.619	0.420	0.342	0.492	0.117	0.465
	*F*_IS_	0.008	0.700	0.096	0.133	0.467	−0.315	−0.178	0.192	−0.191	0.086	0.238	−0.067	0.462
Haemida	Number of alleles	6	3	14	6	2	5	5	4	5	3	6	4	4
	*H*_o_	0.400	0.050	1.000	0.750	0.500	0.550	0.600	0.850	0.600	0.450	0.500	0.300	0.200
	*H*_e_	0.680	0.521	0.890	0.790	0.500	0.623	0.649	0.696	0.576	0.359	0.658	0.306	0.270
	*F*_IS_	0.412*	0.904*	−0.124	0.051	0.000	0.116	0.075	−0.221	−0.041	−0.254	0.240	0.020	0.259

An asterisk on *F*_IS_ indicates significant deviation from Hardy–Weinberg equilibrium at *P *< 0.05 after sequential Bonferroni correction (Rice 1989).

### Population structure

**Figure 3:**
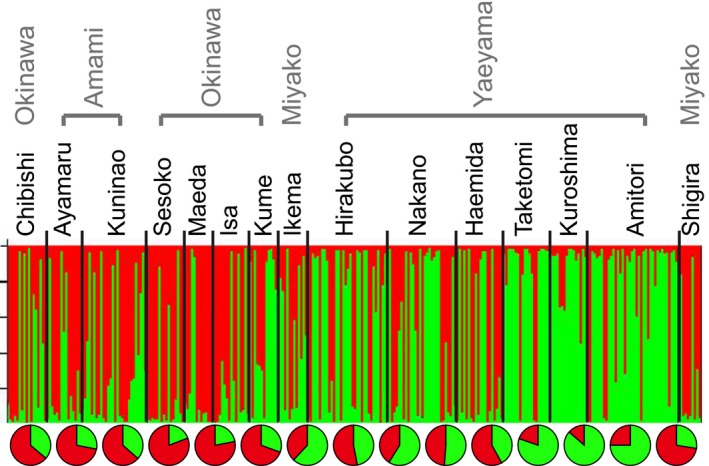
There are at least two inferred populations of *Acropora tenuis* in the Nansei Islands, based on 13 microsatellite loci from 298 individuals. Estimated population structure and ancestral membership coefficients for all 298 individuals, using *K* = 2. Individuals are represented by thin vertical lines, which are partitioned into estimated ancestral population group membership fractions. The sold black line separates sampling locations. Pie charts show the proportions of both populations at each sampling location.

Using the web‐based program, STRUCTURE HARVESTER, we determined that the most suitable number of populations in the study area was two (*K* = 2; Appendices S4 and 5 shows results for *K* = 3, and *K* = 4). These results suggest that the Nansei Islands have two mixed populations that co‐occur in different ratios in different locations (Fig. 3).

**Figure 4:**
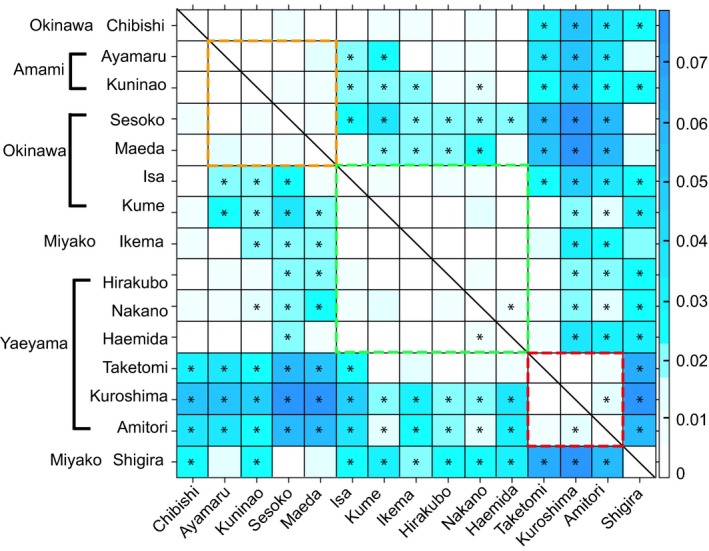
Three rough genetic clusters (dashed squares) were distinguished based on pairwise *F*_ST_ values among 15 sampling locations of *Acropora tenuis*. The heatmap color code denotes the magnitude of *F*_ST_ values (right). The combination with an asterisk was significant (*P* < 0.05, AMOVA, Appendix S6).

Genetic variation among sampling locations is low (= 0.076, 2% of total), but significant (*P* = 0.001), with most of the total variation (4.043) occurring within localities (AMOVA). Genetic variation between localities was generally low (pairwise *F*_ST_'s between locations were low, ranging from 0 to 0.079); nonetheless, 56 of 105 pairs of locations differed significantly (*P* < 0.05; Appendix S6). We distinguished three clusters of *A. tenuis* in the Nansei. These are discernible in a heatmap based on pairwise *F*_ST_ values (Fig. 4). The first cluster consisted of the Amami Islands (Ayamaru [1] and Kuninao [2]) and the northern Okinawa Islands (Sesoko [3] and Maeda [4]; Figs. 2, 4). The second cluster consisted of the central Okinawa Islands (Isa [5] and Kume [7]), northern Miyako Island (Ikema [8]), and some of the Yaeyama Islands (Hirakubo [10], Nakano [13], and Haemida [15]; Figs. 2, 4). *Acropora tenuis* in Chibishi (6), the Kerama Islands shared between the first and second clusters (Figs. 2, 4). Ongoing gene flow from both clusters can be inferred at Chibishi (6) from this gradient in allele frequencies. The third cluster consisted of part of the Yaeyama Islands (Taketomi [11], Kuroshima [12], and Amitori [14]; Figs. 2, 4). Taketomi (11) was shared between the second and third clusters (Figs. 2, 4). The area south of Miyako Island (Shigira [9]) did not differ significantly from Ayamaru (1), the northern Okinawa Islands (Sesoko [3], and Maeda [4]), but it did not cluster with other locations (Figs. 2, 4). Moreover, pairwise *F*_ST_ values from Amami Island did not differ significantly from those of Miyako Island and the southern Yaeyama Islands (Hirakubo [10], Nakano [13], and Haemida [15]; Fig. 4). For the entire data set, no significant correlation (*R* = 0.082, *P* > 0.05) was found between pairwise *F*_ST_ values and geographic Euclidean distance, based on the isolation‐by‐distance model (Appendix S7). It infers that geographically restricted gene flow does not generate a genetic structure in the Nansei.

### Migration rate

**Figure 5:**
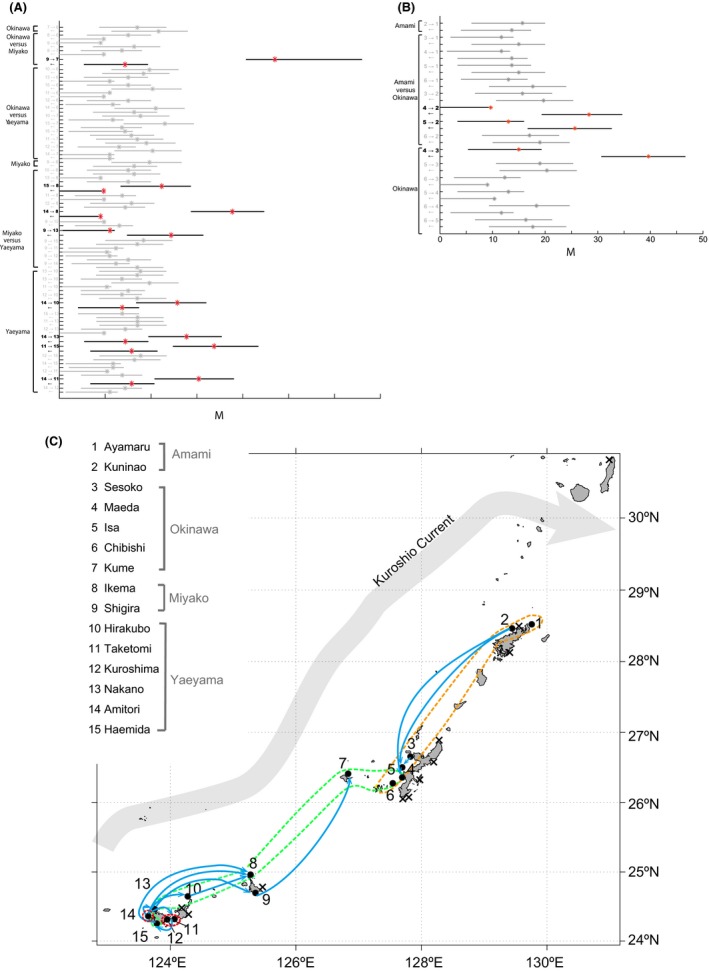
Gene flow in *Acropora tenuis* in the Nansei Islands does not simply follow the Kuroshio Current. Estimates of migration rates between sampling locations for the southern (A) and the northern Nansei (B). In the southwestern Nansei, geneflow is from southwest to northeast; however, in the northern Nansei the reverse is true. In the vicinity of the Sekisei Lagoon, in the extreme southwestern Nansei, gene flow is roughly circular and counterclockwise. The asterisk on each bar represents the median posterior value over all loci. Each bar represents values from the lower to upper quartile (25–75%). The *x*‐axis shows the mutation‐scaled migration rate *M* = *m*/*μ*, where *m* is the immigration rate per generation among sampling locations, and *μ* is the mutation rate per generation per locus. Asymmetric combinations with greater than a 10‐M difference in opposing migration rates are shown with black lines and red asterisks. (C) Blue arrows represent directions of gene flow based on Bayesian inference, but are not meant as specific routes. Only asymmetric combinations with greater than 10‐M differences in opposing migration rates are shown as arrows. Regions enclosed with dashed boundaries (orange, green, and red) indicate three rough genetic clusters in Figure 4.

First, we tried MIGRATE‐N 3.6.11 for all 15 sampling locations, but calculations did not converge. For confirmation, we employed a sister program LAMARC 2.0 (Kuhner 2006) using the recommended default parameters. LAMARC likewise failed to calculate *F*_ST_ values for all sampling localities. According to the LAMARC website, this may have been caused by a case in which intrapopulation variability exceeded that between populations (http://evolution.genetics.washington.edu/lamarc/documentation/index.html). Therefore, migration rates were calculated separately for the northern, central, and southern parts of the Nansei using MIGRATE‐N 3.6.11 (Figs. 2, 5A and B, and Appendix S8A). In addition, because pairwise *F*_ST_ values between the Amami Islands and some locations in the southern Nansei (Ikema [8], Hirakubo [10], Nakano [13], and Haemida [15]) did not differ significantly, we calculated their migration rates (Appendix S8B). Most migration rates were symmetric between pairs of locations (Fig. 5A and B, and Appendix S8A and 8B); however, migration rates for several combinations were strongly asymmetric, indicating much greater gene flow in one direction (Fig. 5A and B, and Appendix S8B). The asymmetric combinations, in which opposing migration rates differed by more than 10‐M, were categorized as having a high rate of gene flow. For example, in the northern Nansei, gene flow from north to south (from Kuninao [2] to Maeda [4], from Kuninao [2] to Isa [5], and from Sesoko [3] to Maeda [4], respectively) was much greater than from south to north (Fig. 5B). In the central Nansei, migration rates are essentially symmetric (Appendix S8A). In contrast, in the southern Nansei, gene flow from south to north (from Shigira [9] to Kume [7], from Amitori [14] to Ikema [8], from Amitori [14] to Hirakubo [10], from Amitori [14] to Nakano [13], respectively) was much greater than from north to south (Figs. 2, 5A). Around Sekisei Lagoon, gene flow from west to east (from Amitori [14] to Taketomi [11]) and from the northeast to the southwest (from Taketomi [11] to Haemida [15]) was much greater than in the reverse direction; this pattern forms a roughly clockwise circle (Figs. 2, 5A). Between Amami Island and some of the southern Nansei, migration rates were not much different (Appendix S8B). Altogether, our migration rate estimates demonstrate that coral larvae are not supplied in a single, uniform direction throughout the Nansei, but that complex regional supply patterns are created locally (Figs. 2, 5C).

### Ocean currents

We extracted 4047 current vectors from the Japan Oceanographic Data Center database, from a region covering the Nansei Islands. This region was divided into a grid containing roughly 200 squares based upon increments of 0.5° latitude and longitude. Some squares lacked current vectors, while most contained several to many. Those within each square were then averaged, resulting in 164 mean current vectors (Appendix S9).

## Discussion

### Genetic diversity

Contrary to studies of other *Acropora* species in Caribbean reefs (Tunnicliffe 1981; Baums et al. 2006), the Great Barrier Reef, and coastal sites along northern Australia (Ayre and Hughes 2000), it seems unlikely that *A. tenuis* propagates asexually in the Nansei, as all colonies showed heterogeneity. The low likelihood of asexual reproduction in the Nansei was previously suggested by allozyme electrophoresis (Nishikawa et al. 2003). It was also demonstrated in East Africa (van der Ven et al. 2015) and at offshore sites in northwest Australia, using microsatellite markers (Underwood 2009). In Australia, a large contribution of asexual reproduction was detected using seven microsatellite loci (Underwood 2009). While most of our sampling sites were also coastal sites, no clonal colonies were detected using 13 microsatellite loci. This might be due to differences in the number of microsatellite markers used or to environmental conditions between Okinawa and Australia. In the Nansei, *A. digitifera* also showed no tendency to propagate by fragmentation (Nakajima et al. 2010). According to an experimental study to determine fragment attachment times, fragments of three congeneric species required 16–24 days to attach to new substrata after stabilization with epoxy (Guest et al. 2011). Under natural conditions in the Nansei with comparatively strong water currents, it may be difficult for *A. tenuis* fragments to stay in one place long enough to reattach.

High inbreeding coefficients (*F*_IS_) and a high frequency of null alleles were found at several loci and sites (Table 2, Appendix S2). Such heterozygosity deficits and the presence of null alleles have been commonly observed for a number of benthic marine organisms (Johnson and Black 1984; Hare et al. 1996; Castro et al. 2006; Lemer et al. 2011), including corals (Underwood et al. 2007; Polato et al. 2010). Thus, it is possible that there exist null alleles in the *A. tenuis* data set. Microsatellite markers, including null alleles, sometimes reduce the sensitivity of estimates of genetic differentiation within and between sites, and they may create false homozygotes. However, Dakin and Avise (2004) showed that frequencies of null alleles below 20% might not affect population genetic analyses. Furthermore, AMOVA, migration models, and even in parentage assessments are less affected by the presence of null alleles when levels of gene flow are high (Dakin and Avise 2004; Chapuis and Estoup 2007) and null alleles lead to slight (only 0.2–1%) reductions in statistical power of STRUCTURE (Carlsson 2008). Departures from HWE were estimated in GENEPOP (Table 2), and null alleles were calculated in MicroChecker in this study (Appendix S2), but a recent study claimed that commonly used null allele detection methods have low reliability (Dabrowski et al. 2014). For confirmation, we tested clone diversity and the most suitable number of populations using STRUCTURE and STRUCTURE HARVESTER without two loci 7961m4 and 4546m2, in which null alleles were detected at most of study sites. Removing these two alleles did not affect the results: Number of multilocus genotypes/numbers of samples = 294/298, the most suitable number of populations was *K* = 2. Consequently, we used all loci throughout our analyses, because there were any missing data that we failed to amplify due to the presence of null allele homozygotes among the 298 specimens. Moreover, Amos (2006) claimed that for some purposes, null alleles may be more informative than normal microsatellite alleles.

Genetic diversity of animals and plants is generally inversely related to latitude (Eckert et al. 2008; Adams and Hadly 2013). However, in the Nansei, genetic diversity of *A. tenuis* does not vary significantly with latitude. In addition, genetic diversity of two congeners, *A. digitifera*, in the Nansei (Nakajima et al. 2010), and *Acropora solitalyensis* Veron and Wallace, 1982 in eastern Australia (Noreen et al. 2013), also does not decrease at high latitude. Considering these results, it is possible that this tendency is common to acroporid corals.

We propose two hypotheses to explain this phenomenon. The first is that the high likelihood of immigration between adjacent localities prevents latitudinal differences. Genetic diversity has been reported not to decline in peripheral populations of vertebrates (Garner et al. 2004) and plants (Gapare et al. 2005) that have low genetic differentiation among localities. The second hypothesis is that the presence of physical boundaries unrelated to organismal habitat preferences, such as strong ocean currents, prevent latitudinal differentiation. For example, between the Osumi Islands and the Amami Islands, the Kuroshio Current acts as a dispersal barrier to various marine organisms, including the Japanese turban shell (Kojima et al. 2000), the luminous marine ostracod (Ogoh and Ohmiya 2005), and the Blacktip Grouper (Kuriiwa et al. 2014). The Kuroshio Current also acts as a boundary for coral and fish species, separating the “southern Japan coastal region” and the “Okinawan region” (Fukuda et al. 1991). Species composition and the number of species in coral communities change drastically at the margin of the Kuroshio Current in the Tokara Straits (Nishihira and Veron 1995; Sugihara et al. 2015). This may also be the case in *A. tenuis*, which is rarely found north of the Tokara Straits, that is, Tanegashima Island. Reproductive effort notwithstanding, if distribution is limited by a physical boundary, the population in question is probably not a true peripheral population.

### Gene flow

Among two sexual reproduction modes of corals, spawning species are considered to have greater dispersal potential than brooding species, based on the length of the competency period of planktonic planulae larvae and on environmental factors (Ritson‐Williams et al. 2009). Based on the results of our STRUCTURE analysis, there are at least two genetic populations of *A. tenuis* in the Nansei. There is no clear geographic break between them and the ratio of the two clusters in each population gradually shifts, indicating their admixed origin. The high level of admixture at *K* = 4, indicates that there is no clear reproductive isolation between these genetic populations (Appendix S5). In addition, ranges without significantly different *F*_ST_ values show that the dispersal range of most of *A. tenuis* larvae is up to several tens of kilometers (Underwood 2009). This is not common among acroporid corals. In the case of *A. digitifera*, Nakajima et al. (2010) suggested that the Nansei population, tested by microsatellite analysis, consists of one genetic group. Nishikawa (2008) also detected a similar tendency in *A. digitifera*, using allozyme analysis. Hence, it is unlikely that a physical barrier exists between the southern and central Nansei. In addition, different patterns of gene flow within the same reproductive mode have often been interpreted as a reflection of larval competency periods (Ben‐David‐zaslow and Benayahu 1998). However, Nishikawa and Sakai (2005) pointed out that this is not always a valid interpretation. They observed that *A. digitifera* had higher gene flow, despite having a shorter maximum competency period (54 days) than *A. tenuis* (69 days). Reasons for different population structures among species include the following: population size, reproductive power, density of mature colonies relative to fecundity, and adaptability to environmental changes. Furthermore, Suzuki et al. (2012) inferred that distribution patterns were determined by segregation of swimming larvae rather than by natural selection after random settlement. Further, *A. tenuis* and *A. digitifera* have significantly different preferences regarding settlement depth (Suzuki et al. 2011) and habitat (Suzuki et al. 2012). Although studies of other species are needed, differentiation of population structure between these species in the Nansei reflects preferences of swimming larvae for settlement sites, rather than different competency periods, and physical habitat attributes, such as water currents and reef structures.

The Amami Islands in the northern Nansei and some locations in the southern Nansei (Miyako Islands, Hirakubo [10], Nakano [13], and Haemida [15]) are not significantly different (Fig. 4). However, if we assume a larger number of cryptic populations, for example, *K* = 4, the composition of the presumed ancestral populations in the Amami Islands is different from that of the Yaeyama Islands (Appendix S5). Moreover, MIGRATE‐N was unable to estimate gene flow between the Amami Islands and other localities in the southern Nansei (Appendix S8B). These results suggest that the microsatellites that we analyzed may not have enough resolution to detect differences between them. More high‐resolution markers, for example, whole‐genome SNPs, might be needed to reveal more detailed population relationships, probably reflecting repeated historical events, such as local extinction and recovery.

On the whole, our results suggest that the Yaeyama Islands and the Amami Islands are potential source populations. On the other hand, the Miyako Islands, the Kerama Islands, and central western Okinawa Island are potential coral sink populations. In the southern Nansei, corals are recruited from south to north, as previously thought, due to the Kuroshio Current. Inside Sekisei Lagoon, migration data indicate a southwestward migration (Fig. 5A and C). This tendency was also shown in a surface water circulation model during the coral spawning season (Lu et al. 2010). In the northern Nansei, *A. tenuis* disperses from north to south. The southward return current along northern Okinawa Island during the middle of the spawning season of *A. tenuis* in May, from 1990 to 2014 also suggests the likelihood of southerly gene flow (Appendix S9). In addition, the geostrophic current from the Amami Islands to Okinawa Island, which flows into the Tokara Straits in opposition to the Kuroshio Current, has been reported (Konaga et al. 1980; Veron and Minchin 1992). Although it seems to have generally been ignored in coral studies to date, we hypothesize that this countercurrent west of the Amami Islands and Okinawa Island has contributed to the establishment of *A. tenuis* in west central Okinawa Prefecture. Additionally, our pairwise *F*_ST_ values suggest two potential contact points for the two genetically different clusters. The first is around Chibishi (6), in the Kerama Islands, and west central Okinawa Island. The second is around Taketomi Island (11). The Kerama Islands are not the only source population for Okinawa Island, but they may have an interdependent relationship.

*Acropora tenuis* has formed the present distributional patterns in the Nansei based upon habitat preferences, complex ocean currents, and geographic and historical features. Genetic compositions of populations have continuously shifted in the process of distributional transition. Consequently, gradual but significantly different genetic populations have formed. These cryptic populations are very important for preserving genetic diversity and should be maintained. Black (1993) noted the higher probability of recruiting coral larvae to the reef of origin. In the case of *A. tenuis*, restoration of coral communities should not rely solely on recruitment from healthy remote coral reefs, but should also conserve local populations and local habitats.

## Data Accessibility

Microsatellite primer sequences are available in GenBank (AB915217–AB915230) and Shinzato et al. (2014).

## Conflict of Interest

None declared.

## Supporting information


**Appendix S1.** Three combinations of multiplexed primer sets developed for this study with fluorescent tails and tagged primers.
**Appendix S2.** Estimated frequencies of null alleles with significant values in bold.
**Appendix S3.** Two parameters of genetic diversity, allelic richness (Ar) and expected heterozygosity (*H*_e_) for each sampling location. These did not differ significantly between locations (Kruskal–Wallis test).
**Appendix S4.** Analysis with STRUCTURE (Pritchard et al. 2000), suggested that there are two probable populations (*K* = 2) in the Nansei.
**Appendix S5.** Estimated population structure using STRUCTURE (Pritchard et al. 2000) for 3 or 4 populations.
**Appendix S6.** Analysis of molecular variance (AMOVA) for multilocus pairwise *F*_ST_ estimates for 13 microsatellite loci from *Acropora tenuis*.
**Appendix S7.** Genetic distance is unrelated to Euclidian distance among Nansei populations of *Acropora tenuis*.
**Appendix S8.** Summary of estimated directions of gene flow for *Acropora tenuis* in the Nansei Islands.
**Appendix S9.** Complex local ocean currents in May in the Nansei Islands, during the *Acropora tenuis* spawning season.
