# Supplementary material for: Unexpectedly complex gradation of coral population structure in the Nansei Islands, Japan
Source: Ecol Evol. 2016 Jul 12;6(15):5491–505. doi: 10.1002/ece3.2296 (PMC4984520; doi:10.1002/ece3.2296)
Supplement: Supplementary file 1 — Appendix S1. Three combinations of multiplexed primer sets developed for this study with fluorescent tails and tagged primers. Appendix S2. Estimated frequencies of null alleles with significant values in bold. Appendix S3. Two parameters of genetic diversity, allelic richness (Ar) and expected heterozygosity (H e) for each sampling location. These did not differ significantly between locations (Kruskal–Wallis test). Appendix S4. Analysis with STRUCTURE (Pritchard et al. 2000), suggested that there are two probable populations (K = 2) in the Nansei. Appendix S5. Estimated population structure using STRUCTURE (Pritchard et al. 2000) for 3 or 4 populations. Appendix S6. Analysis of molecular variance (AMOVA) for multilocus pairwise F ST estimates for 13 microsatellite loci from Acropora tenuis. Appendix S7. Genetic distance is unrelated to Euclidian distance among Nansei populations of Acropora tenuis. Appendix S8. Summary of estimated directions of gene flow for Acropora tenuis in the Nansei Islands. Appendix S9. Complex local ocean currents in May in the Nansei Islands, during the Acropora tenuis spawning season. [file ECE3-6-5491-s001.pdf]

Appendix S1. Three combinations of multiplex primer sets developed for this study with fluorescent tails and tagged primers.

	Tag		
	6-FAM	CAG	T7
combination1	441m6	12406m3	11543m5
combination2	8346m3	7961m4	12130m5
combination3	530m4	11292m4	11745m3

Appendix S2. Estimated frequencies of null alllels with significant values in bold.

	Ayamaru	Kuninao	Sesoko	Maeda	Kume	Isa	Chibishi	Ikema	Shigira	Hirakubo	Nakano	Taketomi	Amitori	Kuroshima	Haemida
8346m3	0.006	0.083	-0.119	-0.069	-0.064	0.108	0.020	0.021	0.149	0.016	0.016	0.091	0.019	-0.001	**0.205**
7961m4	**0.304**	**0.392**	**0.306**	**0.437**	**0.439**	**0.329**	**0.301**	**0.194**	-0.103	**0.349**	**0.237**	**0.411**	**0.294**	**0.273**	**0.387**
11745m3	-0.063	-0.067	-0.038	0.036	0.060	0.046	-0.019	0.030	-0.040	-0.012	**0.073**	-0.001	0.378	0.051	-0.065
12406m3	0.072	0.097	-0.126	0.009	0.000	0.101	-0.009	-0.031	-0.065	-0.067	0.032	-0.093	-0.071	0.070	0.027
11543m5	-0.115	-0.294	-0.138	0.207	0.077	-0.134	0.187	0.160	-0.005	**0.240**	**0.193**	0.054	-0.097	0.207	0.000
530m4	-0.179	-0.124	0.044	-0.264	0.035	-0.044	**0.250**	-0.100	-0.368	0.050	0.022	0.022	0.041	-0.168	0.040
11401m4	-0.072	-0.040	-0.039	**0.182**	-0.022	0.080	0.014	-0.191	-0.105	0.027	0.043	-0.189	0.093	-0.084	0.031
441m6	0.029	-0.012	-0.061	**0.226**	-0.147	-0.228	0.180	-0.027	0.086	**0.106**	0.031	-0.018	0.005	0.111	-0.122
11292m4	0.097	**0.195**	0.108	0.084	0.074	-0.030	0.057	0.025	**0.257**	**0.156**	**0.138**	**0.258**	0.056	-0.260	-0.026
8499m4	-0.177	-0.409	-0.388	0.159	-0.236	0.000	-0.045	-0.499	-0.344	-0.101	-0.232	-0.060	-0.275	0.041	-0.251
7203m5	0.136	0.132	-0.073	0.068	0.027	0.119	**0.205**	0.037	0.079	-0.028	-0.063	0.002	**0.117**	0.137	0.132
12130m5	-0.184	0.001	-0.370	-0.440	-0.087	-0.293	-0.263	-0.119	-0.215	-0.172	0.008	-0.129	-0.054	-0.065	0.041
4546m2	**0.273**	**0.282**	**0.170**	**0.236**	0.029	0.155	**0.270**	**0.234**	**0.288**	**0.243**	**0.105**	**0.268**	**0.142**	**0.209**	0.096

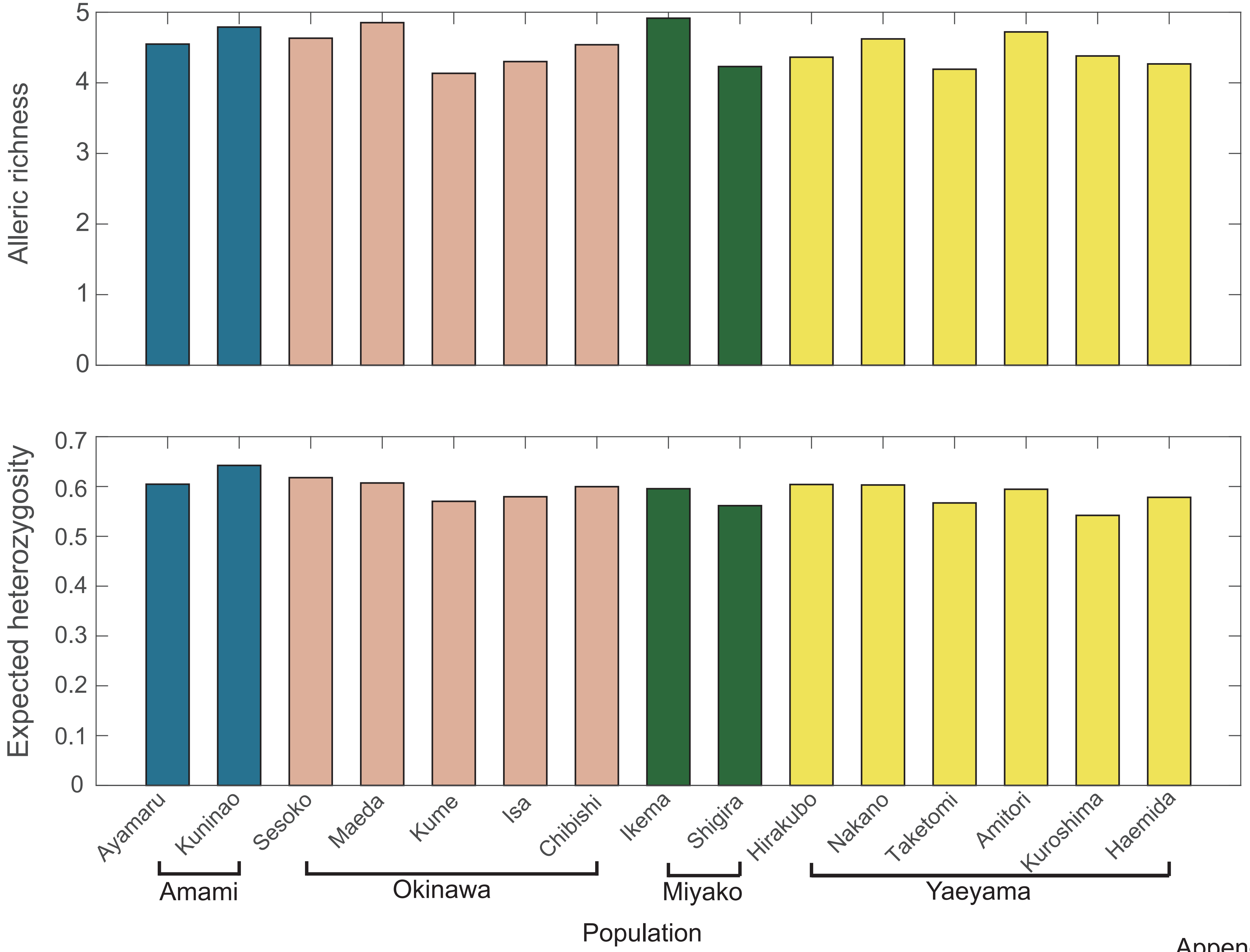

Alleric richness
0
1
2
3
4
5
Expected heterozygosity
0
0.1
0.2
0.3
0.4
0.5
0.6
0.7
Ayamaru
Kuninao
Sesoko
Maeda
Kume
Isa
Chibishi
Ikema
Shigira
Hirakubo
Nakano
Taketomi
Amitori
Kuroshima
Haemida
Amami
Okinawa
Miyako
Yaeyama
Population

Appendix S3

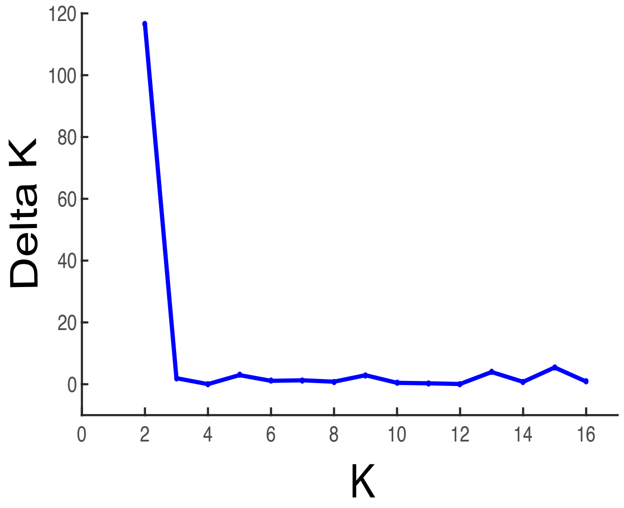

Appendix S4

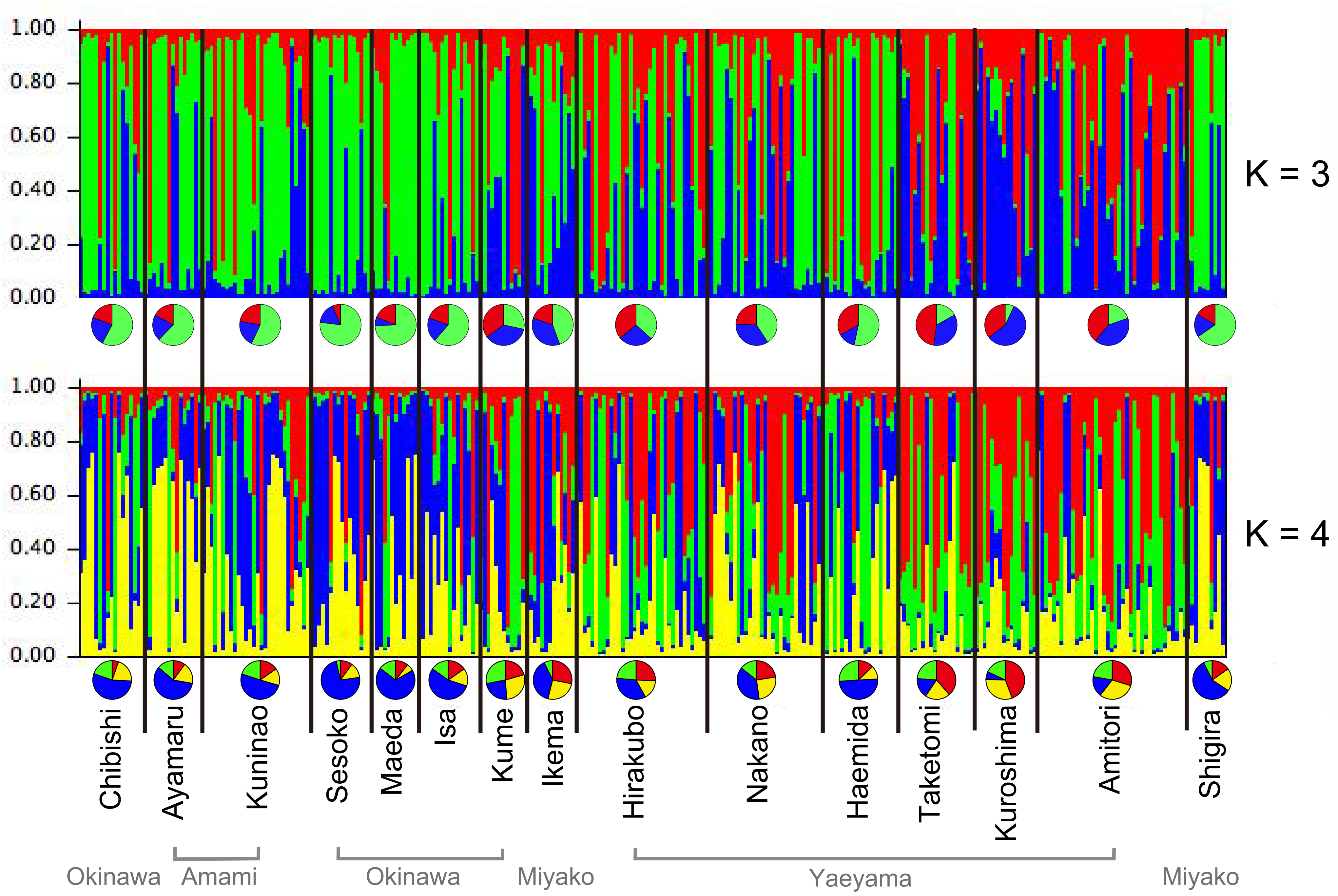

1.00
0.80
0.60
0.40
0.20
0.00
K = 3
K = 4
Chibishi
Ayamaru
Kuninao
Sesoko
Maeda
Isa
Kume
Ikema
Hirakubo
Nakano
Haemida
Taketomi
Kuroshima
Amitori
Shigira
Okinawa
Amami
Okinawa
Miyako
Yaeyama
Miyako

Appendix S5

Appendix S6. Analysis of molecular variance (AMOVA) for multilocus pairwise F_{ST} estimates for 13 microsatellite loci from *Acropora tenuis*.

Chibishi	Ayamaru	Kuninao	Sesoko	Maeda	Isa	Kume	Ikema	Hirakubo	Nakano	Haemida	Taketomi	Kuroshima	Amitori	Shigira	
	0.269	0.422	0.148	0.446	0.344	0.120	0.165	0.171	0.141	0.432	**0.009**	**0.001**	**0.001**	**0.024**	Chibishi
0.004		0.447	0.425	0.068	**0.030**	**0.014**	0.446	0.106	0.095	0.322	**0.002**	**0.001**	**0.001**	0.104	Ayamaru
0.000	0.000		0.116	0.183	**0.008**	**0.010**	**0.019**	0.056	**0.014**	0.348	**0.003**	**0.001**	**0.001**	**0.011**	Kuninao
0.008	0.000	0.008		0.209	**0.014**	**0.006**	**0.035**	**0.015**	**0.008**	**0.016**	**0.001**	**0.001**	**0.001**	0.436	Sesoko
0.000	0.015	0.006	0.007		0.241	**0.047**	**0.044**	**0.021**	**0.007**	0.140	**0.001**	**0.001**	**0.001**	0.137	Maeda
0.002	0.017	0.019	0.023	0.006		0.158	0.446	0.076	0.066	0.439	**0.012**	**0.001**	**0.002**	**0.037**	Isa
0.011	0.028	0.021	0.037	0.019	0.010		0.435	0.349	0.080	0.269	0.254	**0.044**	**0.038**	**0.022**	Kume
0.008	0.000	0.018	0.019	0.019	0.000	0.000		0.305	0.403	0.459	0.100	**0.003**	**0.004**	0.061	Ikema
0.005	0.008	0.008	0.017	0.018	0.009	0.001	0.003		0.071	0.453	0.179	**0.006**	**0.002**	**0.007**	Hirakubo
0.006	0.010	0.014	0.020	0.027	0.009	0.012	0.000	0.006		**0.046**	0.058	**0.019**	**0.005**	**0.023**	Nakano
0.000	0.003	0.002	0.021	0.009	0.000	0.004	0.000	0.000	0.010		0.086	**0.002**	**0.002**	**0.009**	Haemida
0.029	0.034	0.026	0.057	0.053	0.023	0.005	0.012	0.005	0.010	0.009		0.445	0.133	**0.001**	Taketomi
0.056	0.054	0.050	0.079	0.075	0.047	0.018	0.032	0.018	0.018	0.036	0.000		**0.022**	**0.001**	Kuroshima
0.036	0.039	0.024	0.057	0.062	0.038	0.015	0.025	0.018	0.016	0.030	0.006	0.015		**0.001**	Amitori
0.023	0.015	0.024	0.000	0.013	0.024	0.032	0.017	0.026	0.023	0.031	0.065	0.077	0.062		Shigira

F_{ST} values are under diagonal. P-values are above diagonal and boldfaced values are significant ($P < 0.05$).

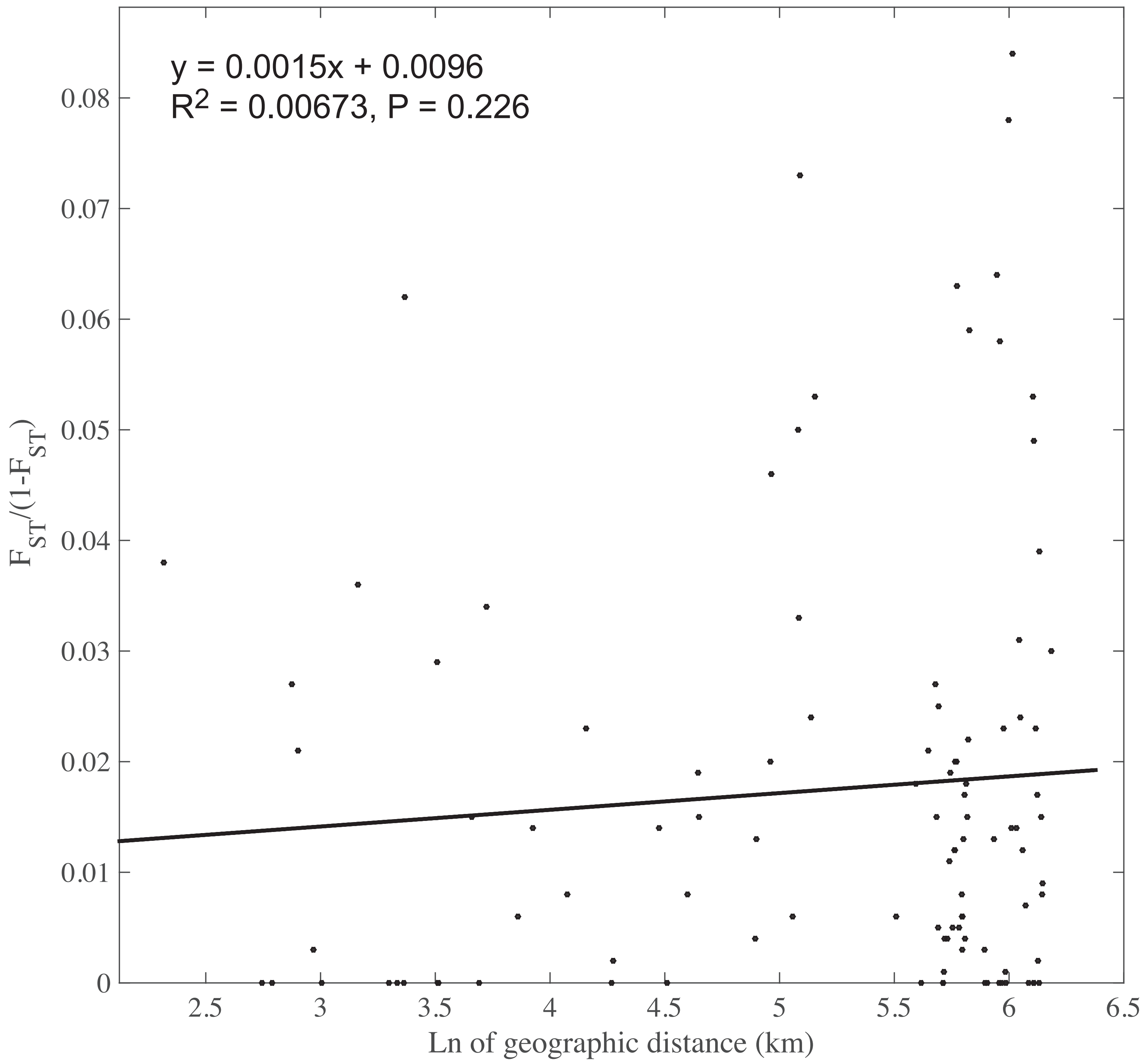

y = 0.0015x + 0.0096
R2 = 0.00673, P = 0.226
0.08
0.07
0.06
0.05
0.04
0.03
0.02
0.01
0
FST/(1-FST)
2.5
3
3.5
4
4.5
5
5.5
6
6.5
Ln of geographic distance (km)

Appendix S7

(A)

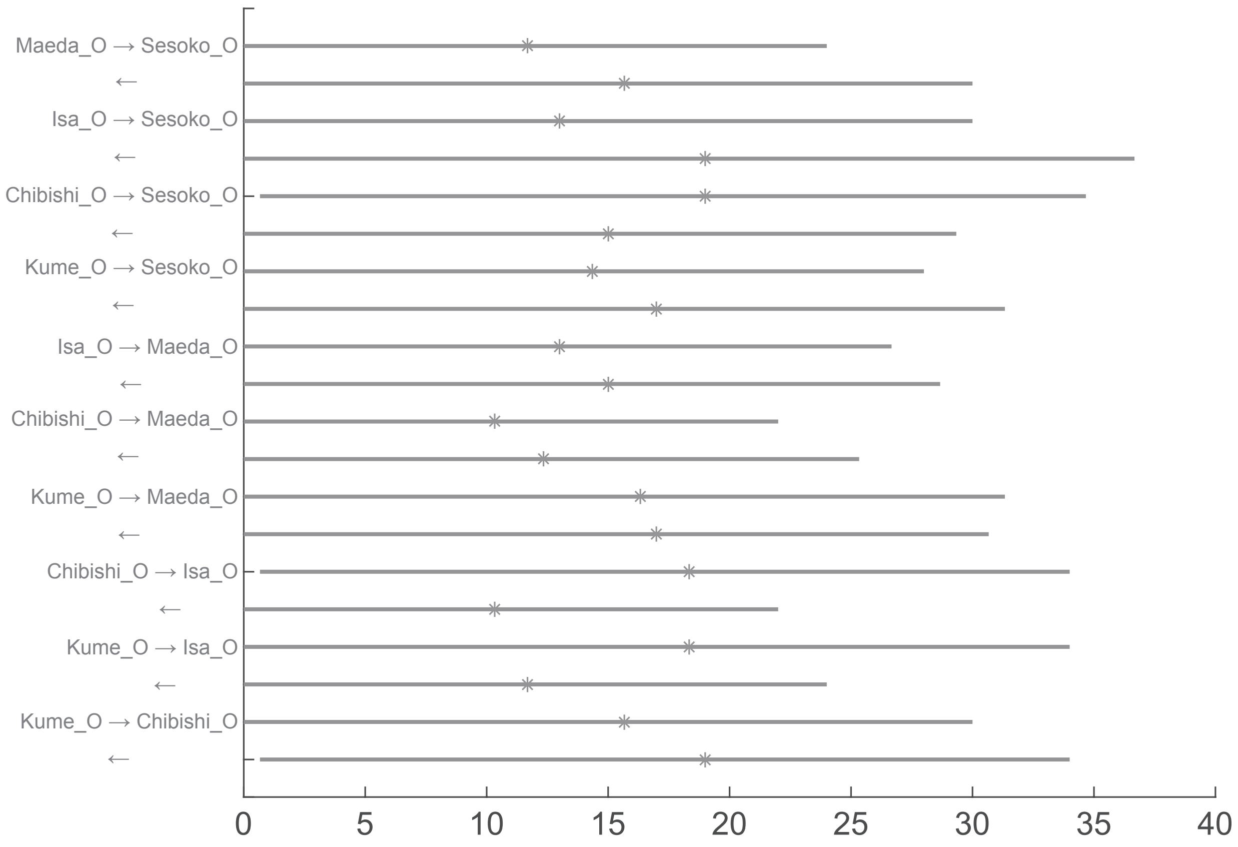

(B)

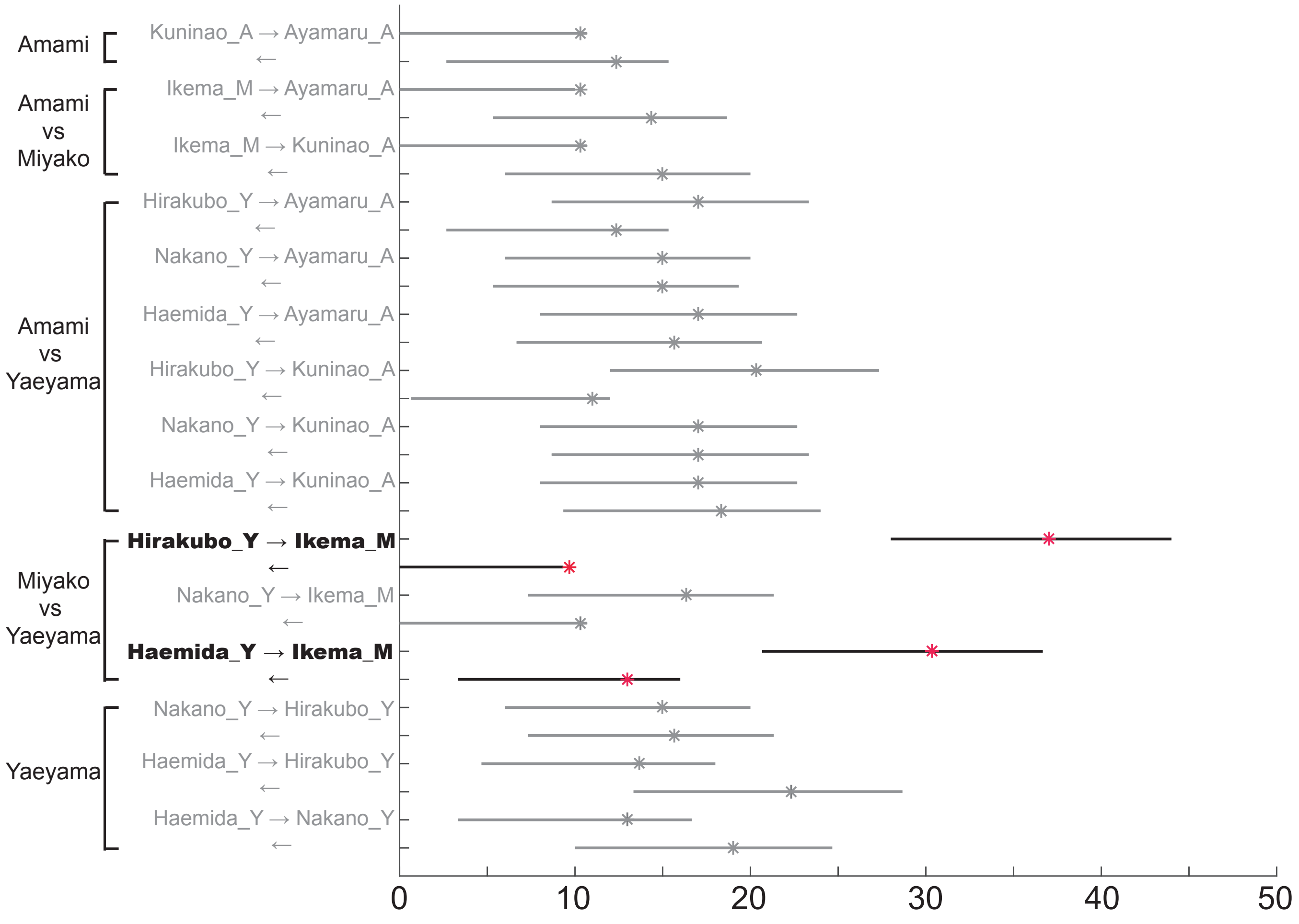

Appendix S8

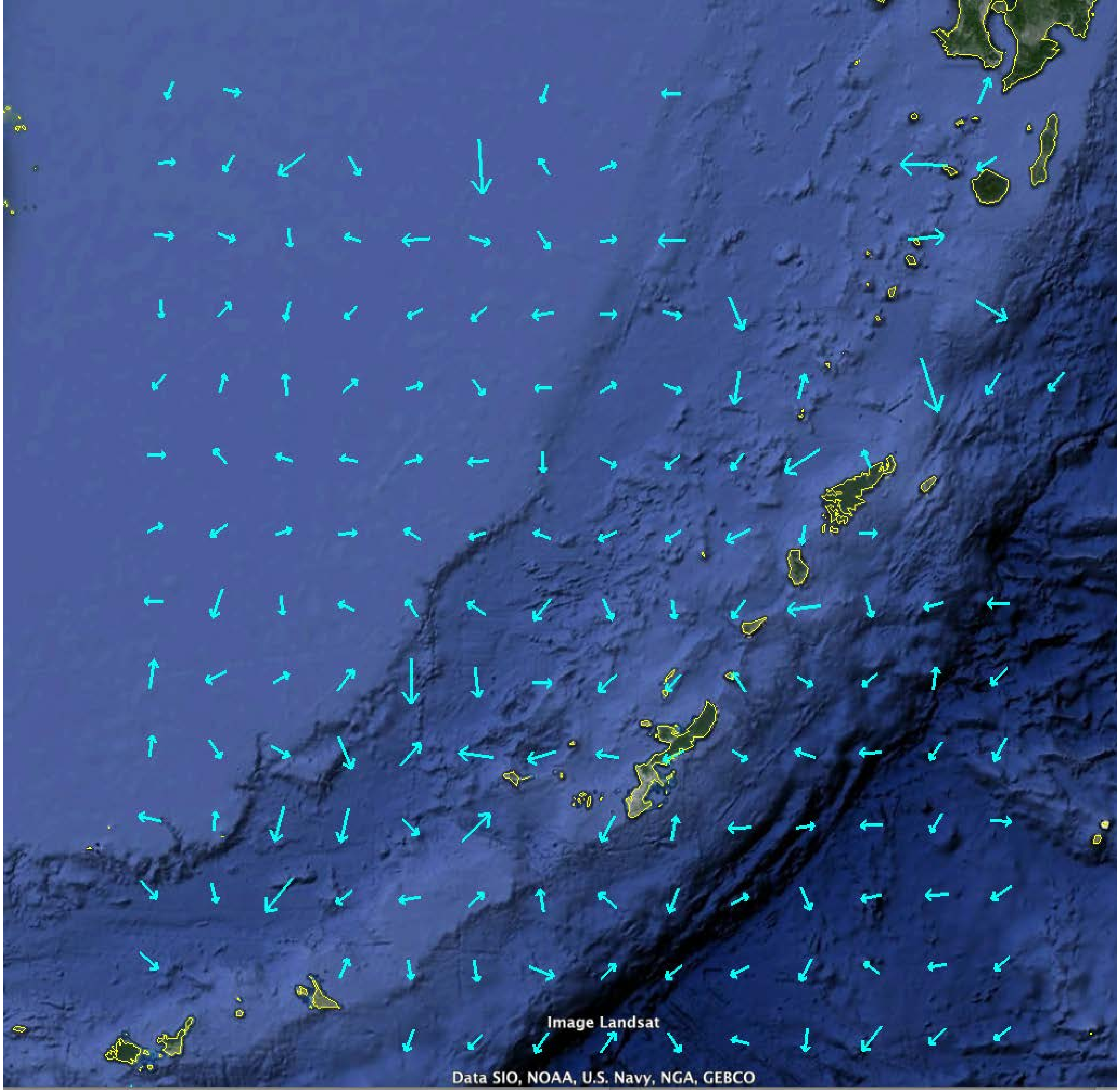

Appendix S9
